# Seroprevalence of foot and mouth disease virus infection in pigs from Zuru, Nigeria

**DOI:** 10.14202/vetworld.2015.865-869

**Published:** 2015-07-14

**Authors:** L. U. Fakai, O. O. Faleke, A. A. Magaji, E. B. Ibitoye, B. R. Alkali

**Affiliations:** 1 Zonal Veterinary Clinic, Kebbi, Nigeria; 2 Department of Veterinary Public Health and Preventive Medicine, Faculty of Veterinary Medicine, Usmanu Danfodiyo University, Sokoto, Nigeria; 3 Department of Theriogenology and Animal Production, Faculty of Veterinary Medicine, Usmanu Danfodiyo University, Sokoto, Nigeria; 4 Department of Veterinary Microbiology, Faculty of Veterinary Medicine, Usmanu Danfodiyo University, Sokoto, Nigeria

**Keywords:** foot-and-mouth disease virus, immunochromatography, Nigeria, pigs, seroprevalence

## Abstract

**Aim:**

This study was conducted to determine the seroprevalence and distribution of foot and mouth disease virus (FMDV) infection in pigs from Zuru, Kebbi State, Nigeria.

**Materials and Methods:**

Cross-sectional serological surveys were conducted between May and December 2013 using the immunochromatography assay technique. Structured questionnaires were administered to households identified at pig rearing areas to obtain the population structures and some information on managemental practices.

**Results:**

A total number of 849 pigs were enumerated at 37 pigs rearing households. Tudun wada had the largest concentration of pigs (237 pigs), while Dabai has the least (38 pigs). A total of 250 blood samples were collected, of which 45 (18%) were positive; Zango has the highest seroprevalence (1.6%), while Dabai recorded the least (0.4%). Based on sex and age, the infection was higher in female (10.4%) and young pigs (11.6%) than male (7.6%) and adult pigs (6.4%), respectively. There was no significant (p>0.05) association between infection and pig rearing areas, sex, and age. Furthermore, none of the 250 pigs examined for classical FMDV lesions was positive.

**Conclusion:**

The results of this study showed that FMD is an important disease of pigs in the study areas. This result justifies the need for more attention and subsequent molecular study to identify the circulating FMDV in the area, which will help in the implementation of effective control measures.

## Introduction

Foot and mouth disease (FMD) is a viral and highly contagious disease affecting cattle, pigs, sheep, goats, buffalo [1-3], and artiodactylous wildlife species [4,5]. It is caused by a positive sense, single-stranded RNA virus, of the genus Aphthovirus, family Picornaviridae [6], of which there are known seven distinct serotypes O, A, C, South African Territories (SAT 1), SAT 2, and SAT 3, and Asia 1 [7]. Infection with any of the serotype does not confer immunity against another [8]. The disease is presented with fever, loss of appetite, salivation, characteristic lesions include vesicles, epithelial erosions and ulcers of the snout, tongue, hard and soft plates, skin of the interdigital spaces, and coronary bands of the feet and teats [9,10]. These signs might vary from mild to severe, although adult animals generally recover; it frequently causes high mortality in young animals [11].

FMD has an almost worldwide distribution with an endemic and occasionally epidemic occurrence. FMD is a highly contagious transboundary animal disease and causes significant economic losses by its high morbidity and the export trade restrictions imposed on infected countries. The disease is mainly spread by direct and indirect contact, mechanical transfer of droplets from infected to other susceptible animals is involved in the former, while the latter is through contaminated vehicles, personnel, and fomites [7]. Airborne transmission over long distances has been reported under favorable climatic conditions, particularly in respect to domestic pigs which is capable of exhaling the highest amounts of airborne virus [12]. This is easily passed onto highly susceptible in-contact ruminants through the respiratory route. In disease-free countries, considerable amount of money is spent on prevention, while endemic countries spend most of their resources on control measures [11]. However, FMD is still endemic in two-third of the world countries and periodically re-emerging in several other countries [11,13]. In most parts of Africa, FMD outbreaks are often not reported either due to its endemicity or the fact that mortalities are low in the infected adult of susceptible animals [14]. In Nigeria, since the livestock population are from both within the country and neighboring countries of west and central Africa, the animals are at high risk of infection of endemic strains as well as antigenic variants prevalent in neighboring countries. Between 1924 and 1981 on a different occasions serotypes O, A, SAT 1, and SAT 2 were identified in outbreaks that occurred in some parts of Nigeria. However, a recent analysis conducted between 2007 and 2009 have shown that O, A, and SAT 2 serotypes are still prevalent [15]. Pigs play a major role in the spread of FMD through their ability to emit airborne FMD virus (FMDV) and cattle are highly susceptible to infection by inhalation [12].

In Nigeria, the current trend of the disease occurrence is unknown but alarming, as there are regular outbreaks, no control measures, poor enforcement of legislation guiding disease reporting to veterinary authority as well as poor control of animal movements [14]. Cattle are most susceptible to infection by FMD followed by sheep while goats remain the least [14], however, pigs are equally highly susceptible [16]. The semi-intensive system of animal management in developing countries encourages the spread of the disease [17]. Furthermore, raising pigs with other domestic animals may increase the risk of infection because they are equally susceptible to FMD and can spread FMDV via aerosol [18]. Once an animal is infected, the virus can be disseminated into the environment including pastures, water sources, and soil. Furthermore, there are no published works on the status of FMD in domestic animals including pigs in the study area and there are only few detailed survey of its prevalence in other food animals, mostly in northern parts of Nigeria. In the study, pig husbandry over time has been on the increase in the number of households due to its cheap management, quick market weight gain, and high reproduction rate. Pig management practice is largely semi-intensive along with other livestock such as cattle, sheep, and goats as well as with dogs and poultry, this can predispose to the easy spread of infection among pigs and other animals. Therefore, it is imperative to assess the current status of FMD infection of pigs in the study area.

## Materials and Methods

### Ethical approval

Ethical Clearance as approved by the Research Ethical Committee of the Faculty of Veterinary Medicine, Usmanu Danfodiyo University Sokoto was obtained prior to this study.

### Study area

The study area Zuru Kebbi State, Nigeria, lies between latitude 11° 5’ to 11° 56’ N and longitude 4° 3’ to 5° 25’ E, it falls within Sudan savannah and has mean annual rainfall of about 600 mm - 1000 mm and mean minimum and maximum temperatures are 27°C and 39°C, respectively [19]. The human population is estimated at 165,547 people, of which 82,941 were males and 82,606 were females with a land area of 653 km^2^ [20]. People in the study area engage in crop farming, hunting, blacksmithing, livestock rearing such as cattle, sheep, goat, and pigs. Pigs are raised semi-intensively and serve as a source of income for the households [19].

### Study design and sampling method

The study was a cross-sectional serological survey to detect antibodies to FMDV in pigs. A pilot survey was conducted using a semi-closed-open ended questionnaire to estimate population, obtain some sociological parameters and information on management systems, and associated risks factors of households pigs raised at Amanawa, Bedi, Dabai, Zango, Tudun Wada, Rikoto, and Senchi of Zuru Territory. Pigs were sampled as they were available, as the owners do not have control over them.

### Sample size determination

The sample size was calculated at 20% prevalence, at 95% confidence interval, with a desired absolute precision of 5% using the formula,


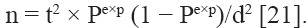


Where n = sample size, t = confidence interval at 95%, P = prevalence at 20%, d = level of precision at 5%. A minimum of 245 sample size was derived and was adjusted to 250.

### Sample collection

Following proper restraint, pigs were aged by teeth eruption, sex was determined by the observation of external genitalia [22] and individual pigs were examined for clinical signs of FMD. About 5 ml of blood each was collected either from the ear vein, mammary gland vein or recurrent tarsal veins of live pigs [22]. Sera were harvested using decantation and centrifugation at 5,000 rpm for 10 min, the sera were then transported in a cold chain container to the laboratory of Veterinary Public Health, Usmanu Danfodiyo University Sokoto, Nigeria for the assay to screen for the presence of non-structural protein antibodies (NSP Ab) to FMDV in a time frame of not more than 1-week.

### Immunochromatographic assay

A commercial NSP kit, Quicking^®^ FMDV NSP Ab Rapid Test, from Quicking Biotech Co., Ltd., Shanghai, China, was used since the kit is known to have a high sensitivity (94.2%) and specificity (98.5%). It is a sandwich lateral flow immunochromatographic assay for qualitative detection of FMD NSP Ab in the serum of all domestic animals including pigs. The test was used according to the manufacturer’s instructions; the cassette was removed from the pouch and placed horizontally. By gradual dropping, 3 drops of the serum was added into the hole “S”, and the results were interpreted between 5 and 10 min. Results after 10 min were considered invalid. The presence of both “C” and “T” bands on the cassette shows a positive result, the presence of only clear “C” band indicates a negative reaction while the absence of any color band on “T” and “C” indicates invalid results.

### Statistical analysis

The results were presented in the form of tables, charts, and percentages. Chi-square (*χ*^2^ – test) was also used to analyze for any significant association between the occurrences of FMDV NSP Ab with pig rearing areas, age, and sex.

## Results

The questionnaire survey conducted shows a total of 7 pig rearing areas, 37 pig rearing households, and a total of 849 pigs comprising both adults and young pigs. Tudun Wada had the highest population of pigs (237) while Dabai had the least (38) (Table-1). All the 250 pigs physically examined for clinical signs of FMD were negative. As shown in Table-2, of the 250 blood samples analyzed for antibodies to FMDV, 45 were positive giving an overall prevalence rate of 18%. No significant association exists between infection and pig rearing areas, sex, and age The prevalence was, however, highest in: Zango (28.57%), followed by Rikoto (24.32%) and Bedi (22.22%), female pigs (19.55%) and pigs under 1-year (21.17%) as shown on Tables-2, 3 and 4, respectively.

**Table-1:** Pigs population by household in Zuru L.G.A of Kebbi State (May-Dec 2013).

Area	Number of pig households (%)	Total number of pigs (%)
Amanawa	4 (9.8)	190 (22.4)
Bedi	4 (9.8)	104 (12.2)
Dabai	2 (4.9)	38 (4.5)
Zango	3 (7.3)	81 (9.5)
Rikoto	6 (14.6)	92 (10.8)
Senchi	10 (24.4)	107 (12.6)
Tudun Wada	8 (19.5)	237 (28.0)
Total	37	849

**Table-2:** Prevalence of FMD infection in Pigs in Zuru L.G.A. Kebbi State (Nov-Dec, 2013).

Area	Number sampled	Number of positive samples	Percentage positive
Amanawa	54	11	20.37
Bedi	36	8	22.22
Dabai	38	1	2.63
Zango	14	4	28.57
Rikoto	37	9	24.32
Senchi	29	5	17.24
Tudun Wada	42	7	16.66
Total	250	45	18

*χ*^2^=8.846, p=0.1824, p>0.05, FMD: Foot and mouth disease

**Table-3:** Sex Distribution of FMD Infection in Zuru L.G.A Kebbi State (May-Dec 2013).

Sex	Number sampled	Number of positive samples	Percentage positive
Male	117	19	16.24
Female	133	26	19.55
Total	250	45	18.0

*χ*^2^=0.2649, p=0.6068, p>0.05

**Table-4:** Age distribution of FMD infection in Zuru L.G.A Kebbi State (May-Dec 2013).

Age	Number sampled	Number of positive samples	Percentage
Under 1 year (<1-year)	137	29	21.17
Above 1 year (>1-year)	113	16	14.16
Total	250	45	18.0

*χ*^2^=1.613, p=0.204, p>0.05

## Discussions

This is the first study to examine the seroprevalence of antibodies against FMDV in pigs in Zuru, and it demonstrates the usefulness of structured surveillance and collecting samples from households’ pigs. The overall seroprevalence of 18% recorded in this study is higher than 3.4% reported by Bronsvoort *et al*. [23] in a study comprising 58 pigs that were managed semi-intensively with other livestock (ruminants) in Adamawa province of Cameroon. The rate is, however, close to 13.6% reported among 116 pigs under semi-intensive management in the remote town of Bhutan, Australia [24]. The high seroprevalence recorded in this study might be due to the large pigs rearing areas covered, considerably large sample size compared to the previous reports, difference in geographical locations as well as degree of association between pigs and ruminant coupled with the 5.9% prevalence rate of FMDV in cattle in border towns of Niger State [25]. This could also be attributed to poor FMD control efforts that include non-vaccination in pigs. Furthermore, the transhumance such as seen in pastoralism where animals move from north to south in search of pasture during dry season and migrate back at the beginning of the raining season can aid the importation of the virus to the study area [26,27].

This research, however, contradicted a report by Lazarus *et al*. [14], who reported 0% seropositive in 90 pigs sampled in Kaduna, North Central Nigeria. This could be due to the fact that pigs sampled in Kaduna were reared under an intensive system of management, thus creating a barrier between the pigs and other livestock. Routine vaccination, prompt medical attention, and environmental hygiene being observed on commercial farms may have to some extent exclude infections from such farms.

In this study, no significant variation was found in the prevalence of FMD between areas. The animal level seroprevalences recorded in three areas of Zango (28.57%), Rikoto (24.32%), and Bedi (22.22%) can be considered high. The possible reason for this could be that Zango is on the fringe of the Zuru emirate while Rikoto and Bedi are the immediate entry and exit routes into and from Zuru Central. Therefore, there could be a possibility of dissemination of this virus on the way to and from the surrounding areas.

No significant difference (p>0.05) was observed in the prevalence of FMD between female and male pigs in this study. This finding was consistent with the previous findings in cattle in Ethiopia [27,28], where sex appeared not to have a significant effect on seropositivity for FMD.

In terms of age, pigs under a year (<1-year) had higher percentage of positive samples than those above 1-year (>1-year), though not significant, this could be attributed to their higher number and also may be because younger pigs are more susceptible and are at high risk of being infected [6,29].

The presence of FMDV NSP antibodies in pigs as observed in this study indicate the possibility that the animals have been exposed to FMDV previously. Further, as per the current understanding, the NSPs are relatively conserved between FMDV serotypes. Absence of clinical manifestation of the disease might be as a result of previous exposure to the FMDV or the strain circulating is not host specific. Therefore, further studies need to be conducted by screening more samples from cattle, sheep and goats, and determine the circulating serotypes in the areas. Pig farmers should be encouraged to adopt new husbandry and management practices to break the chain of disease transmission.

## Authors’ Contributions

This study was part of LUF’s MPVM dissertation. OOF, AAM, and BRA designed, supervised, and approved the study and experimental protocol. EBI performed the statistical analysis and drafted the manuscript while OOF and AAM critically reviewed the manuscript. All authors read and approved the final manuscript.
